# Supplementary material for: Expectations for the Development of Health Technology Assessment in Brazil
Source: Int J Environ Res Public Health. 2021 Nov 13;18(22):11912. doi: 10.3390/ijerph182211912 (PMC8625173; doi:10.3390/ijerph182211912)
Supplement: Supplementary file 1 [file ijerph-18-11912-s001.zip › ijerph-1402779-supplementary.pdf]

Supplementary Material

Article title: Expectations for the Development of Health Technology Assessment in Brazil

Corresponding author:

Julia Simões Corrêa Galendi

Institute of Health Economics and Clinical Epidemiology, the University Hospital of Cologne (AöR)

Gleueler straße, 176 178, 50935, Cologne, Germany

Email: julia.simoes-correa-galendi@uk-koeln.de

tel: +49 (0) 221- 478-30900

List of contents

Table S1. Search strategy for literature review on critical issues in the Brazilian HTA-system

Table S2 Theses and rationale

Table S3 Complete version of the questionnaire

Table S1. Search strategy for literature review.

**Medline search, through PubMed**	**Result**
Health Technology Assessment [tiab] AND Brazil [tiab]	56
Health Technology Assessment [tiab] AND Latin America [tiab]	29
**LILACS search, through VHL**	**Result**
tw:((tw:(technology assessment, biomedical)) AND (tw:(Latin America))) AND (mj:("Technology Assessment, Biomedical"))	21
tw:((tw:(technology assessment, biomedical)) AND (tw:(Brazil))) AND (mj:("Technology Assessment, Biomedical"))	166

VHL: Virtual Health Library, platform for technical cooperation of the Pan American Health Organization (PAHO) for management of health information and knowledge in the Latin America & Caribbean Region. Subject headings have been customized for each database.

Table S2 Theses and Rationale.

Domain	Theses	Rationale
**Education and capacity building**	Thesis 1: In 10 years, Brazil will have sufficient adequately trained experts to conduct HTA studies.	Limited technical and scientific capacity to produce and access the quality of studies has been cited as an impairing factor to the further development of HTA in Brazil and other Middle-income countries. The lack of qualified human resources affects the methodological quality of reports produced and also compromises the capacity to comply with deadlines.
**Public Involvement**	Thesis 2: In ten years, plain public involvement will be guaranteed without compromising the technical quality of the process.	Although public involvement in the HTA process improves the transparency of the HTA agency, one could argue that patients and patient representatives do not have sufficient training and their participation could impact negatively the quality of the final decision. Since 2011, patients, healthcare providers, and manufacturers can participate in online public consultations to express their opinions before the plenary of CONITEC reaches a final decision. However, the incipient diffusion of information for different sectors of society limits the participation of society in the HTA process. Public audiences on the plenary of CONITEC occur only in matters of special relevance.
**Cost-effectiveness analysis (CEA)**	Thesis 3: In 10 years a CEA will be required in the HTA process to obtain coverage of new technologies in the benefits catalog.	According to the Brazilian current legislation, the incorporation of technologies into the Unified Healthcare system must rely on an economic evaluation, although not necessarily a cost-effective analysis. In Brazil, different stakeholders advocate for using cost-effectiveness analysis as an approach to provide the decision-making criteria with transparency and reliability. Although the adoption of cost-effectiveness analysis as a standard part of the process of health technology assessment is supported by

		international experience, the level of importance of the cost-effective analysis in the decision-making process varies, as do the method of interpretation of the results (e.g., cost-effectiveness threshold, efficiency frontier).
**Institutional Framework**	Thesis 4.a: In 10 years, a timeframe of three months to complete the HTA process will be mandatory.	The institutional framework for incorporation of technologies in Brazil was modified along with the substitution of CITEC in 2009 for a Health Technology Assessment (HTA) agency, the National Commission for Incorporation of Technologies (CONITEC). The internal procedures were ameliorated by defining a deadline of 180 days for the appraisal of evidence and the decision-making process that can be expanded to 90 more days. Although the current timeframe represented progress, some countries have a deadline of 3 months for the appraisal of technology.
	Thesis 4.b: In 10 years, the HTA appraisal process for public and private institutions will be merged.	The minimum benefit package of private healthcare insurances is designed based on an independent appraisal process conducted by the National Agency for Supplementary Healthcare (ANS - Agência Nacional de Saúde Suplementar). Although the criteria for incorporation of medical technologies into the private and public health insurances share commonalities, currently the new technologies are obliged to endure two appraisal processes.
**Scope of HTA**	Thesis 5: In ten years, the scope of HTA will be restricted to new medical technologies with high added value (e.g., biologics, biosimilars, combination products, devices, oncologic therapy, among others).	According to the Brazilian legislation within the scope of HTA, drugs, products, and procedures have as main aim to provide healthcare. This definition includes vaccines, diagnostic and therapeutic devices, organizational, informational, and educational systems, programs, and protocols. For the purchase of any medical technology by the public healthcare system, an administrative procedure must take place, including an HTA appraisal. During the first years of CONITEC, assessments of medical technologies with low added value were performed. These technologies were characterized by the low quality of evidence of effectiveness available, high safety because of the long-term clinical experience, and finally by the low budget impact due to depreciation to time, as many were already available in the Brazilian market. Selected international HTA agencies focus only on innovative medical technologies with high added value and high budget impact.
**Methodology of HTA**	Thesis 6: In ten years, the conduction of HTA studies will have a high-quality methodology.	The CONITEC elaborated methodological guidelines on how to conduct HTA studies in order to apply for the appraisal process and have the technology incorporated into the Public Healthcare System (SUS). Although the guidelines oversee the quality of evidence described, some of the reports that applied can be described as having poor methodological quality. The lack of data availability and scarcity of qualified human resources is broadly described as a limiting factor to the development of scientific studies in middle-income countries.
**HTA as the basis for jurisdiction**	Thesis 7.a: In 10 years judicial decisions on individual right-to-health lawsuits concerning the coverage of medical innovative technologies will use CONITEC reports as the basis for jurisdiction	The Judicialization of health care, i.e., the use of legal actions and rights-based injunctions to obtain medical treatments and pharmaceuticals is a complex thematic. The individual right to health lawsuits concern mainly medical technologies that are still to undergo an HTA appraisal or that received a negative recommendation from CONITEC. The majority of these lawsuits are favorable to the citizen and disregard evidence-based information, preventing the government from strategically allocating resources.

Table S3. Complete version of the questionnaire.

**Thesis 1: In 10 years, Brazil will have sufficient adequately trained experts to conduct HTA studies.**				
**This goal will be achieved by the implementation of training courses. The courses should be**	Strongly agree	Somewhat agree	Disagree	Strongly disagree
project-based training	☐	☐	☐	☐
short courses	☐	☐	☐	☐
permanent university-based graduate programs	☐	☐	☐	☐
permanent university-based postgraduate programs	☐	☐	☐	☐
**The courses should be offered by…**	Strongly agree	Somewhat agree	Disagree	Strongly disagree
Private universities	☐	☐	☐	☐
Public universities	☐	☐	☐	☐
Public institutions	☐	☐	☐	☐
International collaborations	☐	☐	☐	☐
Public–private partnerships	☐	☐	☐	☐
**Teaching strategies for these courses should be…**	Strongly agree	Somewhat agree	Disagree	Strongly disagree
In-class traditional courses	☐	☐	☐	☐
Online courses	☐	☐	☐	☐
Combination of in-class and online courses	☐	☐	☐	☐
**I consider the achievement of this thesis as…**	Very probable ☐	Probable ☐	Improbable ☐	Very improbable ☐
**Open-ended questions:**<br>**Do you have comments on the proposed thesis?**<br>**Are there other measures to promote education and capacity building in HTA in Brazil?**				
**Thesis 2: In ten years, plain public involvement will be guaranteed without compromising the technical quality of the process.**				
**I consider the achievement of this thesis as…**	Very probable ☐	Probable ☐	Improbable ☐	Very improbable ☐
**This goal will be achieved through the following measures.**	Strongly agree	Somewhat agree	Disagree	Strongly disagree
Standardized advertising to the public to stimulate participation in public consultations.	☐	☐	☐	☐
Establishing a mandatory non-voting seat for patients´ representatives in the Plenaries of CONITEC.	☐	☐	☐	☐
Establishing a mandatory voting seat for patients´ representatives in the Plenaries of CONITEC.	☐	☐	☐	☐
Training of patients' representatives in HTA to support participation in public consultations	☐	☐	☐	☐

Thesis 3.a: In 10 years a cost-effectiveness analysis will be required in the HTA process to obtain coverage / reimbursement of new technologies in the benefits catalog.				
**This goal will be achieved through the following measures.**	Strongly agree	Somewhat agree	Disagree	Strongly disagree
Implementation of mandatory cost-effectiveness analysis (reinforced by law)	☐	☐	☐	☐
Self-binding from the pharmaceutical industry on producing cost-effectiveness analysis.	☐	☐	☐	☐
Self-binding from the plenary of CONITEC to refuse other types of economic evaluations that are not a cost-effectiveness analysis.	☐	☐	☐	☐
**The cost-effectiveness analyze should be carried out by…**	Strongly agree	Somewhat agree	Disagree	Strongly disagree
Independent public universities staff	☐	☐	☐	☐
Independent private universities staff	☐	☐	☐	☐
Private consultancy institutions	☐	☐	☐	☐
Pharmaceutical industry	☐	☐	☐	☐
An internal capacitated commission of CONITEC	☐	☐	☐	☐
**The interpretation of the results of the cost-effectiveness analyses should use the method of…**	Strongly agree	Somewhat agree	Disagree	Strongly disagree
A cost-effectiveness threshold.	☐	☐	☐	☐
An efficiency frontier approach.	☐	☐	☐	☐
**If a cost-effectiveness threshold is chosen, it should be based on…**	Strongly agree	Somewhat agree	Disagree	Strongly disagree
The value suggested by the WHO for middle-income countries. (3GDP/QALY)	☐	☐	☐	☐
A range of values developed based on precedent decisions made by CONITEC. (1 to 3GDP/QALY)	☐	☐	☐	☐
A standard value to be developed by Brazilian researchers based on the budget available for health in Brazil (or willingness to pay).	☐	☐	☐	☐
**The cost-effectiveness threshold should be applied as…**	Strongly agree	Somewhat agree	Disagree	Strongly disagree
Most important or single criteria for incorporation and reimbursement of new medical technologies.	☐	☐	☐	☐

One criterion among others (including ethical, evidence of medical benefit, health care priority, practicability, etc.)	☒	☐	☐	☐
**I consider the achievement of this thesis as…**	Very probable<br>☐	Probable<br>☐	Improbable<br>☐	Very improbable<br>☐
**Open-ended questions:**<br>**Do you have comments on the proposed thesis?**<br><br>**Are there other measures to promote public participation in HTA in Brazil?**				
**Thesis 4.a: In 10 years, a timeframe of three months to complete the HTA process will be mandatory.**				
**This goal will be achieved through the following measures.**	Strongly agree	Somewhat agree	Disagree	Strongly disagree
By rewriting the specific legislation	☐	☐	☐	☐
by expanding the capacity of NATS	☐	☐	☐	☐
by Expanding capacities and training of the CONITEC	☐	☐	☐	☐
By limiting time for public consultations	☐	☐	☐	☐
**I consider the achievement of this thesis as…**	Very probable<br>☐	Probable<br>☐	Improbable<br>☐	Very improbable<br>☐
**Open-ended questions:**<br>**Do you have comments on the proposed thesis?**<br><br>**Are there other measures to improve the institutional framework of HTA in Brazil?**				
**Thesis 4.b: In 10 years, the HTA appraisal process for public and private institutions will be merged.**				
**I consider the achievement of this thesis as…**	Very probable<br>☐	Probable<br>☐	Improbable<br>☐	Very improbable<br>☐
**This goal will be achieved by the following measures.**	Strongly agree	Somewhat agree	Disagree	Strongly disagree
Mutual acceptance of HTA decisions in the private and public scheme, no matter which committees were responsible for the final determination.	☐	☐	☐	☐
Increase of participation of the ANS in the plenaries of CONITEC and consummate a joint appraisal committee.	☐	☐	☐	☐
Forbidding of an independent HTA appraisal process for private health insurances.	☐	☐	☐	☐
Creating a third regulatory body independent from the ministry of health that deliberates on recommendations either from public or private agencies.	☐	☐	☐	☐

**I consider the achievement of this thesis as…**	Very probable ☐	Probable ☐	Improbable ☐	Very improbable ☐
**Open-ended questions:**<br>**Do you have comments on the proposed thesis?**<br><br>**Are there other measures to improve the institutional framework of HTA in Brazil?**				
**Thesis 5: In 10 years, the scope of HTA will be restricted to new medical technologies with high added value (e.g., biologics, biosimilars, combination products, devices, oncologic therapy, among others).**				
**This goal will be achieved through the following measures.**	Strongly agree	Somewhat agree	Disagree	Strongly disagree
To apply specific legislation to medical technologies with low budget impact (e.g., over-the-counter drugs, organizational and informational protocols, among others).	☐	☐	☐	☐
To allow the CONITEC more discretion on decisions concerning low-budget impact technologies, without an HTA process.	☐	☐	☐	☐
**The decision if an HTA process is mandatory will be based on**	Strongly agree	Somewhat agree	Disagree	Strongly disagree
Public health needs	☐	☐	☐	☐
Frequent right to health lawsuits concerning a specific technology	☐	☐	☐	☐
Unmet medical needs	☐	☐	☐	☐
The fact that the technology was previously not evaluated in other countries	☐	☐	☐	☐
High therapeutic value of the technology	☐	☐	☐	☐
**I consider the achievement of this thesis as…**	Very probable ☐	Probable ☐	Improbable ☐	Very improbable ☐
**Open-ended questions:**<br>**Do you have comments on the proposed thesis?**<br><br>**Are there other measures to improve the scope of HTA in Brazil?**				
**Thesis 6: In ten years, the conduction of HTA studies will have high-quality methodology.**				
**This goal will be achieved through the following general measures.**	Strongly agree	Somewhat agree	Disagree	Strongly disagree
To expand the capacity and training of the CONITEC	☐	☐	☐	☐
To expand the capacity and training of (NATS	☐	☐	☐	☐
To review and update periodically the methodological guidelines on how to conduct HTA studies	☐	☐	☐	☐

To provide financial support to independent researchers from public universities that dedicate themselves to HTA	☐	☐	☐	☐
To provide financial support to independent researchers from private universities that dedicate themselves to HTA	☐	☐	☐	☐
To expand the capacity of data collection of "DATASUS"	☐	☐	☐	☐
To ameliorate the accessibility of "DATASUS"	☐	☐	☐	☐
To implement mandatory integrated electronic databases in the main hospitals	☐	☐	☐	☐
To implement mandatory integrated electronic databases in centers of the "Family Health Program"	☐	☐	☐	☐
**I consider the achievement of this thesis as…**	Very probable ☐	Probable ☐	Improbable ☐	Very improbable ☐

Open-ended questions:
Do you have comments on the proposed thesis?

Are there other measures to improve the methodology of HTA in Brazil?

Thesis 7.a: In 10 years judicial decisions on individual right-to-health lawsuits concerning the coverage of medical innovative technologies will use CONITEC reports as the basis for jurisdiction

**This goal will be achieved through the following measures**	Strongly agree	Somewhat agree	Disagree	Strongly disagree
Establishing expert groups to advise the law courts regarding HTA.	☐	☐	☐	☐
Forbidding Judges to challenge HTA decisions that were made by CONITEC.	☐	☐	☐	☐
Ruling that in each lawsuit counseling by CONITEC or by the Ministry of Health is mandatory for the court.	☐	☐	☐	☐
Narrowing timeframe for HTA appraisal to 3 months to ensure timely CONITEC reports.	☐	☐	☐	☐
Implementing an early awareness system (or horizon Scanning) to identify innovative medical technologies with the potential to become a target of lawsuits.	☐	☐	☐	☐
Making HTA appraisal by CONITEC mandatory after ANVISA grants authorization	☐	☐	☐	☐

to enter the market to ensure timely CONITEC reports.				
**I consider the achievement of this thesis as…**	Very probable<br>☐	Probable<br>☐	Improbable<br>☐	Very improbable<br>☐
**Open-ended questions:**<br>**Do you have comments on the proposed thesis?**<br>**Are there other measures to improve the judicial endowment of HTA in Brazil?**				

Abbreviations. HTA: health technology assessment; CONITEC: National Commission for incorporation of Technologies; NATS: nuclei for HTA; GDP: gross domestic product; QALY: quality-adjusted life year; CEA: cost-effectiveness analysis; CET: cost-effectiveness threshold; ANS: National Agency for Supplementary Healthcare; ANVISA: National Health Surveillance Agency; DATASUS: Information Technology department of the National Healthcare system.
